# Understanding global perspectives for the acceptance of community sanitation welfare schemes through a localised qualitative survey in Kho Nagorian, Jaipur, India

**DOI:** 10.1038/s41598-024-65191-1

**Published:** 2024-07-11

**Authors:** Vinayak Gupta, Hazel B. Gonzales, Douglas Hungwe, Yamsomphong Kanokwan, Fumitake Takahashi

**Affiliations:** 1 Department of Transdisciplinary Science and Engineering, Tokyo Institute of Technology, G5-8, 4259 Nagatsuta-Cho, Midori-Ku, Yokohama, 226-8502 Japan; 2 Department of Civil and Environmental Engineering, National University of Singapore, 1 Engineering Drive 2, #07-03 E1A, Singapore, 117576 Singapore; 3 Innovator and Inventor Development Platform (IIDP), Tokyo Institute of Technology, J3-18, 4259 Nagatsuta-Cho, Midori-Ku, Yokohama, Kanagawa 226-8503 Japan; 4 Research and Development Center, Hosei University, 4342 Aihara, Machida, Tokyo 194-0298 Japan

**Keywords:** Public welfare schemes, Safe sanitation, Sense of entitlement, Swachh Bharat Abhiyan, Qualitative survey, Cross-table analysis, Psychology, Environmental social sciences

## Abstract

Community sanitation is a fundamental human right and need. Every year, as per the World Bank, total cost of providing sanitation services is estimated at around 114 billion USD per year. In India, Swachh Bharat Abhiyan (SBA), a public welfare scheme (PWS), is aimed at addressing community sanitation problems. Despite the successful implementation of SBA, local communities still practise open defaecation. To deduce the behavioural patterns governing communal toilet use, interviews were conducted with the local communities in the Kho Nagorian area of Jaipur, Rajasthan, India. This qualitative survey examined attitudes towards the construction of a toilet, awareness towards the SBA scheme, and the willingness to use excreta-based pit humus. The study then discusses the factors that increase the local community’s willingness to use these toilets. Results show that open defaecation is still prevalent in society. One way to foster the adoption of toilets is that the construction materials should mainly consist of local materials. As a recourse, places of worship could be used to influence people’s perception of hygiene. In addition, community toilets should be cleaned often as well. PWS should not be made accessible at no cost to prevent a sense of entitlement among the people. A small sum should be charged to increase social responsibility towards the PWS. Another way to curb open defaecation is to tap into the sense of entitlement by making effective use of social campaign programs. Further, cross-table analysis revealed that the locals were inclined to use a toilet if they have invested in it. Advertisements were found to be ineffective, and proposals were made to make them effective. These findings aid in understanding public perceptions and can guide the development of public policies. The findings also assist in making tax distribution decisions that reflect public concerns, attitudes, and values.

## Introduction

Community sanitation is a prerequisite for healthy societies. It relates to public health issues such as safe drinking water, as well as the treatment and disposal of excreta. Sanitation systems are designed to preserve human health by creating a clean environment that prevents disease transmission, mainly via the faecal–oral pathway. This has evolved into a way of life, despite its origins as a means of achieving preventive health-related problems. The principle of sanitation is to transfer all decomposable matter, solid waste, liquid, and gaseous waste from dwellings immediately after production to a designated waste treatment facility. In this sense, a country's sanitary infrastructure development potentially serves as a sensitive indicator of economic growth. While potable drinking water is essential in providing environmental engineering services, the importance of an up-to-date sewerage system cannot be overlooked. It cannot be allowed to fall behind, as all of the water used by the community must flow back as excreta loaded with the waste of community living unless adequately collected, treated, and disposed of; this would create serious water pollution problems^1,2^.

Poor sanitation can cause water pollution problems, and it is an important issue in developing countries^3,4^. Though around $114 billion per year is spent on sanitation services^5^, still, worldwide, an estimated 2.6 billion people live without satisfactory sanitation services^6^. In India, during the 1980s, the rural sanitation coverage was estimated to be 1%^7^. Since 1980, various government programs at the central government, state government, and local municipality levels have resulted in rapid improvements in sanitation and drinking water delivery^7^. Swachh Bharat Abhiyan (SBA), also known as the Swachh Bharat Mission or Clean India Initiative is one such attempt to address the sanitation problems faced by communities in India^8^. It is a country-wide campaign initiated by the government of India in 2014 to eliminate open defaecation and improve solid waste management. The campaign aims to achieve an "open-defaecation-free" country by constructing community toilets. This campaign was divided into several phases, wherein the first phase was completed in October 2019.

Under the SBA, the first phase included eradication of manual scavenging, generating awareness, and bringing about behavioural change regarding sanitation practices, and augmentation of capacity at the local level. Though this campaign has achieved its target to a greater extent^9–12^, open defaecation in slum areas remains prominent^12^. In the country, about 67% of rural and 12% of urban households still practice open defaecation^13^. Despite the successful implementation of SBA, local populations still use manual scavenging and open defaecation. In the 1800s, manual scavenging and open defaecation were mainly due to the caste system^14^. The caste system was banned in 1950 but open defaecation is still prevalent. In addition, recent studies have shown that the rural sanitation sector in India is marked by intense spatial inequality and heterogeneity^13,15^. There have been studies and opinions in academia on what it will take for people to actually use government-subsidized toilets^16,17^. The reasons for those can be understood through public opinion research, which includes target interviews with the direct beneficiaries and the local communities, and by understanding their perspectives. Public opinion research, including highly structured laboratory exercises and observation of people's behaviour, aids in identifying the information needed to address organisational and service issues. It helps in weighing public perception on a specific issue, such as when government contractors call to inquire about access to healthcare or social services, or when there is a need to understand why a community is not using toilets. A survey study also helps in understanding prevalent societal issues, such as problems incurred during the construction of a toilet and the implementation of a government scheme.

In the past, similar contingent valuation surveys have been used to investigate the main variables of excreta reuse, such as water conservation, health and environmental benefits, hesitancy to use wastewater, treatment costs, religious restriction, education, and awareness level^18–21^. These conclusive elements determine whether a wastewater-reuse project will succeed or fail. For example, a study found that the water, sanitation, and hygiene (WASH) practices among the tribal population of Tamil Nadu, India are not acceptable^22^. Similarly, on a global scale, in a study by Kantanoleon et al., a questionnaire was sent to the parents of high school students in Chalkida City, to gauge public opinion on wastewater reuse. The results indicated that the public was optimistic about the industrial application of wastewater reuse (76%)^18^. In a similar study by Marks (2006) food-related uses, such as animal crops and vegetable cultivations, were discussed. In the survey conducted in the United States (47–74% public acceptability) and Australia (> 95% public acceptability), the public expressed strong support for wastewater reuse in industrial sectors, public parks, school grounds, and golf course irrigation^20^. However, the public's main concern was potential health problems associated with recycled water^18,20^. The findings revealed that environmental and socio-economic factors play a significant role in excreta reuse applications^23,24^. Sharing relevant information, on the other hand, can improve public support for the reuse ideas^25,26^. Therefore, efforts should be made to encourage broader thinking among the public for the reuse of excreta^27^.

Through improved communication and involvement, addressing public issues can encourage more productive public dialogue and help to strengthen public confidence and trust. For this reason, the public's perspective was studied to determine people's willingness to reuse treated excreta, and their attitude towards basic hygiene. Consequently, this research adds to recent data on public attitude towards an ongoing public welfare scheme (PWS). In this light, this study conducted a sociological survey to gather the thoughts and perceptions of residents in the slum areas of Jaipur to better understand factors that influence public and consumer perception. To further investigate the relationships between variables such as demographics, behaviour towards the PWS, enthusiasm to pay money for toilet building and product preferences, cross-table analysis has been conducted. A cross-table analysis (CTA) is a statistical technique used to examine the connection between two or more categorical variables^28^. The analysis comprises a table detailing the percentage or frequency of observations for each group of the variables under consideration. The final table is referred to as a cross-tabulation or a contingency table which enables spotting patterns and connections between the variables by demonstrating how the categories of one variable relate to the categories of another variable^28^. CTA is a commonly used method in public health and environmental research. In Ghana, a study applying the CTA to examine disease transmission and the separation between homes and final disposal sites revealed that people living closer to open dump sites were more likely to contract related illnesses like malaria and skin infections^29^. Similarly, the outcomes of this study can be applied to design public benefit schemes in many developing countries around the world. This survey, thus, aims to assist in decision-making regarding tax money allocation by reflecting public concerns, views, and values. In addition, this study also aims at contribute towards target 6.2 of the Sustainable Development Goals Number 6 established by the United Nations in 2015^30^.

## Materials and methods

The study has been narrowed down to the Kho Nagorian slum area in the city of Jaipur. Home to around four million residents^31^, Jaipur is widely celebrated as a famous tourist spot and is a UNESCO world heritage city. This section discusses the sanitation issues in Jaipur and the approach and methodology of the citizen survey used in this study.

### Study area

**Figure 1:**
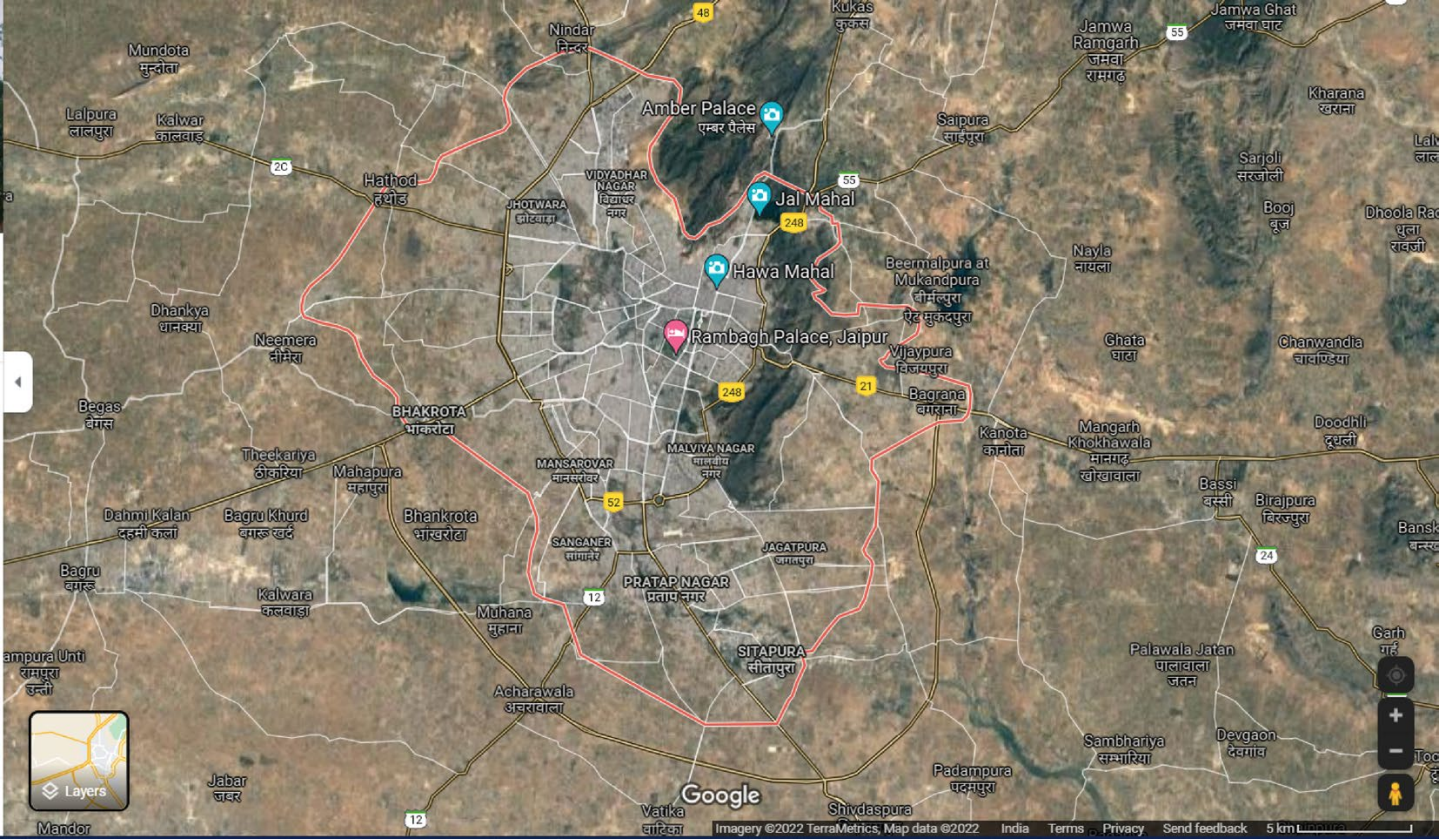
Satellite imagery of Jaipur City (Rajasthan, India) marked under red-coloured boundary (procured from Google maps on 13 January 2022).

**Figure 2:**
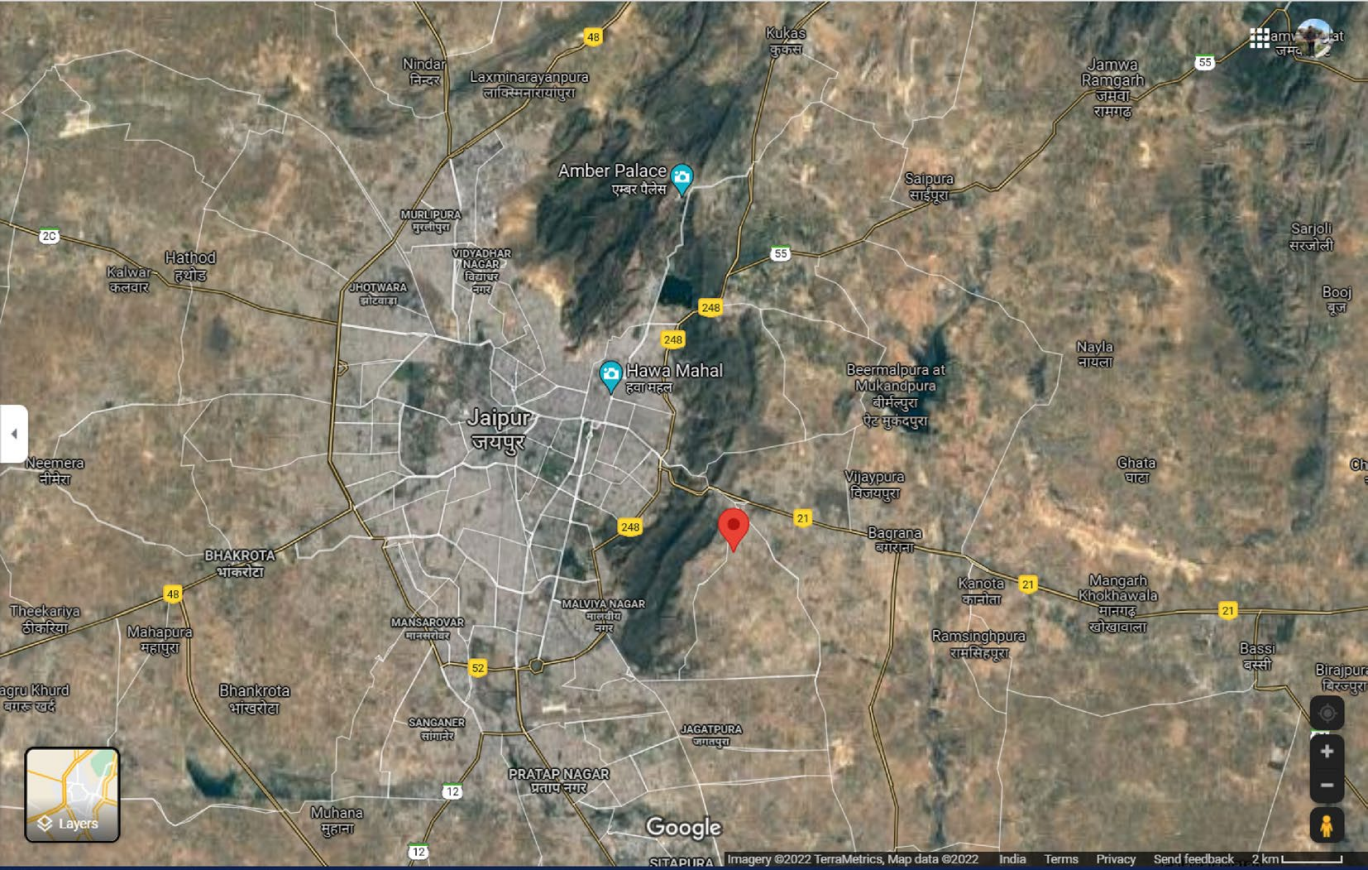
Satellite imagery of Kho Nagorian in Jaipur City marked as a red-coloured indicator (procured from Google maps on 13 January 2022).

Jaipur is the capital and largest city of the Indian state of Rajasthan. The city is 268 kms (167 miles) from New Delhi, the national capital of the country. Jaipur is situated at a latitude of 26° 55ʹ N and a longitude of 75° 49ʹ E, whereas its municipal limit stretches from 26° 46ʹ N latitude to 27° 01ʹ N latitude, and from 75° 37ʹ E longitude to 76° 57ʹ E longitude (Fig. 1). The area of study is a “*kasba*” or locality known as Kho Nagorian which is located at about 13 km SE of the walled city of Jaipur (Fig. 2). This area is dominated by an uneducated class, mostly unskilled labour.

The daily solid waste generation in Jaipur is almost 1100 MT (metric tonnes)/day^32^. In Jaipur, around 200–250 MT is left on the streets and highways, implying an overall lifting efficiency of around 80%. The per capita solid waste creation per day is roughly 350 g, equating to 1.75 kg per day for a family of almost five^32^. The lack of a community garbage collection facility in slums is a common sight wherein slum residents dump their rubbish near their residential areas^33,34^. Due to the onset of SBA, these sites have drastically improved, though there is a long way to complete transformation^35^. Despite efforts, some areas in old Jaipur face overflowing garbage cans and indiscriminate dumping^36^. Burning of waste leads to thick smoke on roads. Many drains and sewers near Mother Dairy, Bais Godam, Durgapura and Pratapnagar remain blocked due to improper waste disposal^33,34^. The lack of convenient excreta disposal facilities and their treatment in less developed areas, including the slum area of the Kho Nagorian, encourage people to use neighbourhood open lands for defaecation.

### Methodology

A collection of preliminary data was acquired using a qualitative survey in a low-income community. After getting the public responses, trends in user behaviour were observed and further CTA has been performed to analyse further relationships between the variables.

#### Collection of preliminary data

Residents' concerns regarding the success of the SBA and their attitude toward the sanitation practices were collected through a qualitative survey and participatory appraisal. The qualitative survey was performed per the Declaration of Helsinki and was approved by the Human Subject Ethics Review Committee at the Tokyo Institute of Technology (permit number 2023118). Informed consent was obtained from all the participants and/or their legal guardian(s) (in cases where the participant was illiterate). The households were chosen randomly from the Kho Nagorian village so most of them do not have a toilet in their residences as of November 2020. The responses were divided into two categories: households with and households without toilets. A random sampling among sixty households from different parts of Kho Nagorian village was carried out, which included not only the men (considered to be the head of the household) but also women. Out of the sixty households, eight households were omitted due to verification problems.

Out of those remaining fifty-two households reported, fifteen households have toilets on their premises. The rest, thirty-seven households, do not have access to a basic sanitation facility. The questionnaire was designed in both English and Hindi, wherein Hindi is the language spoken by individuals. Table 1 is the display of questions asked in the survey.

**Table 1:** Questionnaire in this study.

Question number	Question
1	Why is the individual not using a toilet?
2	Where do the male members go for defaecation?
3	Where do the female members of the household go for defaecation?
4	Where do the kids of the household go for defaecation?
5	Does the individual face any problems due to not having a toilet facility? What are they?
6	Does the individual think having a toilet can significantly improve above mentioned problems? If no, then how?
7	Does the individual intend to pay more expenditure of money than the government's aid for the construction of toilets if it exceeds the subsidy?
8	In case there is no money from the government would the individual still construct a toilet at his/ her own expense?
9	According to the individual, how much money does it cost to construct a toilet?
10	What problems does the individual incur with/while constructing a toilet?
11	How much money is the individual happily willing to pay for a good toilet facility in your house?
12	What does the individual think of toilets which are low in cost and fast to construct?
13	Would the individual be interested in constructing a fast (in some hours) and easy-to-install toilet for his/ her family?
14	What material does the individual think toilets should be made of?
15	Where does the individual see advertisements for toilets?
16	What are the individual’s perceptions regarding using pit humus from the toilet?

The following subjects were considered (refer to Appendix A for the detailed questionnaire):

- Demographic information, including gender, age ranks, contact number and the number of family members
- Behavioural questions about the usage of toilets by family members
- Issues faced while opting for open defaecation
- The attitude of people towards the construction of a low-cost toilet
- Awareness of people towards the SBA scheme
- Willingness to use the pit humus obtained from treating the excreta from the toilet

Out of these fifty-two households, only two households practice the Hindu religion, while it was noticed that the rest of the fifty households were Muslims. Each household had a distinct contact number/ cellular phone number to maintain a unique identity. This did not allow the repetition of answers from the same household. The said on-site survey was conducted in November 2020, to determine public responses to the concept and application of the construction of toilets and the attitude towards safe defaecation. Collected questionnaire forms were imported into Microsoft Excel for further analysis using statistical tools to develop descriptive statistics.

#### Further investigation through CTA

CTA has been performed on the following cases:

- To understand the trend between the awareness of how much the toilet building will cost the respondents and the enthusiasm to construct a toilet, a CTA has been performed on Question 9 and Question 12. Question 9 tests the awareness of the respondents and tells if the people are aware of the toilet building cost. Question 12 gauges enthusiasm in the toilet construction process.
- Question 11 has been cross analysed with Question 16 to understand the trend between investing in a facility, here, a toilet, and using it.
- A CTA between Question 7 and Question 11 will help to understand the intricacies of human consumer behaviour. Question 7 takes note of the hypothetical willingness to pay for the toilets, and, on the other hand, Question 11 elucidate on the actual willingness to pay money for a toilet building. A comparison between them would help to understand if people would pay for the service in real, rather than hypothesizing it, or if they might not want to pay in actuality, but end up paying nevertheless.
- To deduce the efficacy of advertisements, Question 15 has been analysed across Question 11 and Question 12. Question 15 tells if the respondents have come across an advertisement related to sanitation and hygiene or not.

### Ethical guidelines

The study was approved by the Human Subject Ethics Review Committee of Tokyo Institute of Technology through permit number 2023118.

## Results and discussion

This section comprises a discussion of the demographic distribution of the study area. The results of the survey are divided into three sections: (1) the existing attitude of the local community towards defaecation and associated issues, (2) public trust in wastewater management companies and government efforts in wastewater reuse, and (3) the opinions of the local community on treated wastewater reuse. The results of the CTA have been discussed afterward in four subsequent sections.

### Demographic distribution

The demographic data of the respondents is presented in Table 2. Males made up 80.77% of the responders, while females made up 19.23%. The average number of family members is 7.19. Such demographic data aid in defining the population composition, which is vital in understanding acceptance and attitude towards the SBA and excreta reuse initiatives investigated in this survey.

**Table 2:** Demographic summary of the respondents.

Attributes	Distribution	Units
Total number of valid entries	52	Individuals
Number of male respondents	42	Individuals
Number of female respondents	10	Individuals
Age of oldest respondent	73	Years
Age of youngest respondent	30	Years
Average family size	7.19	family members
Lowest family size	3	family members
Highest family size	30	family members

Kho Nagorian is a Muslim-dominated area. High average family size is a typical characteristic of low-income Muslim families, as mentioned by a study conducted by the Pew Research Centre (Washington, D.C) in 2017^37^.

### Existing attitude towards defaecation and issues faced

Question 1 to Question 5 in Appendix A deal with the existing attitude towards defaecation and the issues associated with open defaecation. These questions were asked to thirty-seven families with no existing toilets in their households. These families opted for open defaecation.

Figure B.1 in Appendix B shows that about 89% of the responses declared financial constraints, and about two individuals opted for “other reason”. While one respondent could not argue the said “other reason”, the other mentioned that the landlord did not provide a toilet in the household. About 6% of the respondents, or two households preferred open defaecation due to fresh air and open fields.

As far as places for the open defaecation is concerned, Fig. B.2 in Appendix B compares the preferred places for open defaecation. The respondents are categorized as males, females, and kids. It is observed that there is a similar pattern in all three categories. Most respondents preferred to defaecate in an open *nallah* (an open drain to allow rapid drainage of rainfall or industrial wastewater.) The next preferred option for open defaecation is a nearby hill. The other options are jungle, nearby agricultural lands, lush green lands, and open barren lands. Regarding “other” responses recorded in the survey, one of the household chiefs was too old to understand the questions posed to him, so his response was excluded. This is noted as “others”. A lady has put in her response as a *doongar*, put in as the “other category”. A *doongar* is a nearby hill in local language but has been put in the “others” category to maintain the validity of the responses in the survey. Two families did not report any kid in their households, so their responses in question 4 have been marked under “others”. It is noted that though there were community toilets, families did not use them. This might be due to the prominent two reasons. One, the community toilet was not in their “*dhani*”, or neighbourhood, and two, community toilets were poorly maintained.

In addition, there have been issues faced by the inhabitants while opting for open defaecation which is shown in Fig. B.3. Twenty-five households reported facing harassment from society because of open defaecation. In addition, accounts of illness related to unhygienic practices were reported. Additional responses included encounters with snakes and insects in the jungle and nearby hills. Time constraints and a desire for privacy during defecation were reported as well. The responses in the “others” category were that the children have pathogens in the stomach, there were inconveniences during the rainy season, and potable water was scarce onsite. A respondent said her impaired daughter defecates at home. Three households have reported that they have no issues with open defaecation.

### Public trust in the wastewater management company and institutions' efforts in wastewater reuse

This argument is discussed in the context of Questions 10 and 15 to every household with or without a toilet in their houses. The number of responses, henceforth, is fifty-two. This section is further divided into two sections: understanding the problems incurred during toilet construction including the public trust in the wastewater management company and investigating the role of public institutions to influence the community's behaviour.

#### Understanding the problems incurred during toilet construction and public trust in the wastewater management company

Question 10 tries to understand the problems incurred during toilet construction, and the responses are shown in Fig. B.4 (Appendix B). Thirty-seven respondents felt that there is a lack of funds and resources to construct a toilet. This is followed by a lack of conviction towards the government officials and contractors that the said contractor might do a substandard job while retaining a fraction of the money earmarked for purchasing materials for construction, as shown in Fig. B.4. Two respondents had no idea what it takes to construct a toilet. Question 15 tests the awareness towards the SBA. About twenty-one individuals have seen an advertisement in the government dispensary and eleven households have been informed by their kids as the kids are being advised about toilets in their schools (Fig. B.5). Ten respondents have seen and heard about the toilets on cellular phones and televisions, and six individuals have also seen advertisements on village walls. This number is higher than those who have not seen or heard an advertisement about the SBA or the usefulness of toilets which counts to twenty-four. This value does not indicate a complete absence of advertisements seen within the respondent's household. Rather, it illustrates that only the specific respondent has not been exposed to any advertisement of toilet-building schemes.

#### Investigating the role of public institutions in influencing the community's behaviour

Studies have shown that lower-income individuals hold stronger religious beliefs than their higher-income counterparts^38^. In addition, places of worship can be a suitable setting to encourage public engagement and spread ideas relating to health promotion^39^. In developing countries, this trait of a community can be used to influence the community's behaviour. Here, it is proposed that the religious places of worship, mosques (as far as this study is concerned because 96% of respondents practice Islam), can be used to encourage hygienic practices and inform people about government programmes in areas where technology is not readily available.

As far as SBA is concerned, the government's efforts are commendable^11,40^. As per data, 89.9 million toilets were built after the launch of SBA^41,42^. Individuals are more aware of toilets, and hygienic practices and have seen the advertisements of toilets at various places as compared to pre-2014^43,44^. However, the local fieldwork and the policy-making should be continued to achieve zero cases of open defaecation in these local communities.

### Respondents’ choices and opinions on treated wastewater reuse

The following questions try to understand the choices and opinions of the respondents on the reuse of treated wastewater; Questions 6, 7, 8, 9, 11, 12, 13, 14 and 16 in Appendix A. These questions were asked to the households without toilets, hence the number of respondents in this section is thirty-seven.

#### Respondents’ alignment with toilet adoption but reluctance to pay

Every respondent agreed that having a toilet in their household will elevate problems posed by open defaecation, as a response to Question 6. Academically as well, district-level data for the year 2011 shows the availability of toilets positively impacts the economic well-being of women^45^. In continuance, Question 7 points out that though every respondent agrees that having a toilet is beneficial for their household, not every person wants to contribute more than the government’s aid to construct toilets. Only twenty-seven out of thirty-seven, approximately 73% of the respondents, agreed to participate and pay more than the government aid to construct a toilet. When someone takes part in activities with others, like the SBA and building toilets, it means they are joining in society and showing others how to do the same. Participating in social events makes one feel like the person is a valuable member of society, giving a sense of purpose and belonging^46^. However, in response to Question 8, when asked if the respondents would want to construct a toilet at their own expense, all the respondents disagreed (thirty-seven respondents in total).

Question 9 tests the people's awareness towards the financial constraints of toilet construction, as shown in Fig. B.6 in Appendix B. Twenty respondents had no idea how much the construction costs, followed by a range between 0 to 10,000 INR by nine respondents. People's attitude to willingly construct a toilet is discussed under the pretext of Question 11, and as shown in Fig. B.7. Twenty-seven out of thirty-seven respondents refused to pay anything for toilet construction. The responders exhibited an attitude of entitlement.

#### Perceptions of low-cost toilet construction and participation intentions

Focus has been paid on people's attitudes towards the construction of a low-cost toilet and whether people would participate in the construction process, which is done under the pretext of Questions 12 and 13 (in Appendix A), respectively. About 78% (twenty-nine households) of respondents were hopeful that a low-cost toilet was trustworthy and worked for them if someone could provide a cheaper toilet facility. However, the remaining 22% (eight households) were apprehensive of such a facility citing that such a low-cost toilet implies poor quality as a response to Question 12. It was observed that not many individuals were passionate about contributing towards toilet construction. For example, in response to Question 13, twenty-eight out of thirty-seven respondents did not want to indulge in the toilet construction process while only nine respondents expressed their desire to participate in the toilet construction.

#### Navigating the challenges of sense of entitlement

It was noticed that citizens have self-entitlement in a way that they believe every service should be free of charge or available at no cost. In this study, people wanted to have a toilet in their households, preferably under a government scheme but rarely wanted to either contribute or help in the construction process. It is proposed that one way to deal with the sense of entitlement is to add value to the services provided to the people. Ramit Sethi mentioned that people value what they pay for^47,48^, so PWS should not be free.

Concerning this study, the sense of entitlement can be tapped to motivate people to make their demands for hygiene from the governments and the stakeholders. Communities and citizens can also be motivated through social campaigns^49^. In this case, rather than publishing only about the usage and the health benefits of a toilet by the government, the government can also emphasize that people should have a right to have a toilet in their households and that they should approach government offices for help in building a toilet in their households. In that sense, when entitled individuals assess that they have a right to a service, the citizens might actually make efforts to get their rights.

#### Acceptance probability of toilets based on construction materials

Question 14 assesses the likelihood of acceptance of the toilets constructed from the various types of construction materials. Local materials found in an area were the most preferred option for constructing a toilet (Fig. B.8). In this study, redstone and mudbricks were the readily available and the most preferred material, so they were easily accepted by the locals for toilet construction. Construction of the toilets using local materials would not only increase the acceptance of these toilets but would also strengthen the local economy. The construction sector maintains employment, pays wages, and contributes to local economies^50,51^. As a result, the local economy will grow since the project's workers will have the income to spend at other nearby firms.

#### Public willingness to utilize excreta-derived pit humus

Question 16 studied people's willingness to use the pit humus from the excreta. Twenty-two respondents were comfortable using the pit humus either for all uses or for all uses except for the crops (Fig. B.9, Appendix B). Psychological inhibition of the use of human faecal matter in agriculture has been reported as a big hindrance to the acceptance of pit humus obtained from a toilet.

### Awareness versus enthusiasm in the toilet construction process

Table C.1. in Appendix C shows the results of the CTA between Question 9 and Question 12. As noticed from the table, 16 households or 43.24 percent of the total responses have no idea how much a toilet building costs them, and they are enthusiastic that it works best for them if someone can provide the toilet facility at lower price. On the other hand, only 2 households, or just 5.40 percent of the total responses are enthusiastic about a fast-to-construct toilet. This tells that people are more likely to help in the toilet building process when they have no idea how much a toilet building will cost them.

### Attitude of the respondents towards investment in the toilet building process versus using the toilet in its full capacity

Table C.2. in Appendix C shows that 33 households or 89.18 percent of households have a polarised view on using pit humus. They will either use it or would not use it at all. Only 4 households or 10.82 percent of households are willing to use the pit humus for growing non-human consumption crops only. In addition to this, when the households have invested money in the toilet-building process (rows 2 to 4), they are more inclined to use the pit humus. In that case, 3 households are not willing to use the pit humus, as compared with 7 households who are willing to use the pit humus either fully or with constraints. This is because those who have paid for the services are more inclined to use them because they have invested in them.

Since the people have a personal stake in the service's success, they might be more apt to use it and benefit from it.

### Consumer behaviour towards paying for the toilet building service

Table C.3 displays the relationship between Question 7 and Question 11. 23 households or 62.1% of total responses have opted for a “no” in both the questions, and 7 households or 18.9% of total responses have opted for a “yes” in both cases. This means that 81% of total cases have opted for the same cases in both questions. Those households are either not interested to pay for the toilet services at all or might want to pay for the same. However, there are 3 responses or 8.1% of people who opted for a “yes” in Question 7 but a “no” in Question 11. Hypothetically those households would pay money but, they would prefer not to. The reason could be well engraved in how the human mind works. The human mind is not always logical and is calculative^52^. It seems that those households have not made their decision yet and that there is a second thought behind their minds. There could be a thought that in the future if they can benefit, they can change their minds to paying for the service. Those respondents must have realised that Question 7 is hypothetical, and so have answered that question at that time. Since the respondents are not 100 percent sure of the response, they have had given an answer even if it might not align with their beliefs. These responders must have been under the impression that if a real question comes, they would answer that then.

Four households have declined to pay in the hypothetical question but ended up actually paying for it. People may initially deny paying due to social pressure or expectations. However, when faced with a situation where they can make a difference for someone else, they may feel compelled to pay despite their initial reluctance. As per research, there is a strong and subversive normative message hidden within the statement "Many people are doing this undesirable behaviour", under the effect of social pressure^53^. Normative messages are successful at promoting "desired" pro-environmental behaviour if a majority of people engage in it^54^. To maximise the effectiveness of normative appeals, descriptive norms (what people usually do) and injunctive norms (what people usually approve or disapprove of) must be in alignment^61^. People's motivations and reasons for paying for a service can be complex and multifaceted. Hence, each individual's unique circumstances and perspectives should be considered when trying to understand consumer behaviour.

### Efficacy of the advertisements

Table C.3 analyses the relationship between Question 15 and Questions 11 and 12. Though, in Question 15, the responses are “Government Dispensaries/Hospitals”, “Television/Mobile phones”, “Village walls”, “Schools”, and “Never heard of one”; for comparison, all these responses have been compiled to only two categories: if the respondent has seen an advertisement or if he has not seen any.

Interestingly, in the first analysis of Question 15 with Question 11, people who have never seen an advertisement are more likely to pay for the services (9 households or 42.85% of respondents in that category as compared to 12 households or 57.14% of respondents in that category). Among the households who have seen an advertisement, 2 households or 12.5% of respondents in that category would pay for the service as compared to 14 households or 87.5% who are not interested to pay for the service. In the second analysis wherein Question 15 has been analysed across Question 12, no trend is evident. Though it might be expected that people who saw an advertisement would be more eager to pay for the service or believe that a low-cost toilet would be good for them, the data reveals another set of information. It can be inferred that the advertisements are not as effective as they should be. During the survey, it was observed that the locals are unable to relate to the advertisements and may not understand them due to lack of education. There is also no call to action in the advertisements, leaving people clueless about using the SBA scheme. Though SBA has been proven to be a promising welfare scheme and toilets are found in the localities, there are households who are unaware of this PWS. If more work is done on advertisements, and the findings of this study are taken into account, then those households can be addressed as well. The points raised would not only assist in improving the SBA but would also assist in creating PWS and policies worldwide.

## Conclusion

There has been influx of money and the resources to promote safe sanitation and hygiene worldwide, yet 494 million people still defaecate in the open^55^. This concentrated research has inadvertently brought up some conclusions regarding the attitude of low-income families towards the government schemes of toilet construction.

1. The efforts of the Indian government are commendable under the SBA Scheme. Open defecation still occurs due to poverty and lack of toilets.
2. There are community toilets, but many families do not use them because the toilet is not in their neighbourhood and the community toilets are not maintained well.
3. To increase the acceptance of toilets in low-income communities, locally available construction materials are preferred and accepted. This is also anticipated to strengthen the local economy.
4. Locals in low-income communities display a high sense of entitlement. They want to have a toilet in their households but rarely want to contribute to the toilet-building process or pay for the services.
5. People unaware of how much a toilet building will cost them, are more apt to participate in the construction process.
6. People have a polarised view of using the pit humus obtained from the toilets. They will either use the pit humus in all forms or would not use it at all. There are no intermediate perspectives.
7. Those who have invested in the toilet building process are more likely to use the services fully, including the full utilization of pit humus from the toilets, hence increasing the acceptance of the toilets.
8. There have been instances, though few, when people would pay for the services under social pressure, feeling of guilt or fear.
9. Advertisements related to SBA are ineffective.

### Making urban sanitation more accessible

There have been studies on transforming the behavioural norms of rural populations to revitalize the PWS through depiction (highlighting the dignity and cultural enrichment that toilet facilities can bring to households), demonstration (organizing live toilet demonstrations for easy operation and maintenance), and divulsion (raising awareness about health risks of open defaecation and promoting low-cost toilet construction)^17,56,57^. Adding to these points, through this study, it is proposed that religious places of worship can be used to promote hygiene practices and notify government schemes to the people where technology is not much evident. These PWS should not be accessible at no cost. For example, to reduce the ‘feeling of entitlement,’ a small sum may be set aside for installing toilets. To increase toilet usage in entitled communities, it is proposed to tap into their sense of entitlement by running social campaigns that emphasize having a toilet as a basic right. For a better understanding of wastewater reuse perspectives and acceptability, knowledge and prior experiences, which can be obtained through various information-sharing channels, are essential. The primary means of informing the public is through the media (such as television, radio, and newspapers); nevertheless, the number of educational programmes should be increased. As far as advertisements are concerned, people prefer real-world examples of the product or service to the generic stock photos that everyone else uses^58,59^. Actual pictures could be utilised to stir up an emotional response^60^. In addition, there should be a compelling call to action^61,62^.

### Informing policy formulation and implementation

Experts working in the fields of public health, sanitation and policymaking may find approaches and answers to the technical and scientific difficulties involved with excreta reuse and management engaging and interesting. Such in-depth and subsequent studies contribute significantly to our understanding of human perceptions, which are often ignored when formulating policies or introducing PWS. Furthermore, fixing some of the challenges discussed here would benefit the public's understanding through improved techniques and aid in drafting public policies and schemes.

### Supplementary Information

Supplementary Information.

## Data Availability

The datasets generated and/or analysed during the current study are not publicly available due to the privacy reasons of the subjects, but are available from the corresponding author on reasonable request.
