# Supplementary material for: Understanding global perspectives for the acceptance of community sanitation welfare schemes through a localised qualitative survey in Kho Nagorian, Jaipur, India
Source: Sci Rep. 2024 Jul 11;14:16002. doi: 10.1038/s41598-024-65191-1 (PMC11239806; doi:10.1038/s41598-024-65191-1)
Supplement: Supplementary file 1 — Supplementary Information. [file 41598_2024_65191_MOESM1_ESM.docx]

**Supplementary material for the “Understanding global perspectives for the acceptance of community sanitation welfare schemes through a localised qualitative survey in Kho Nagorian, Jaipur, India”**

**Appendix A: Customer Survey in Open defaecation Localities, Jaipur**

**LOTA**- **Understanding Target Audience**

**Name /नाम**-                                                                                                                **Age** /**उम्र**–

**Address /पता-**

**Number of members in family /परिवार में सदस्यों की संख्या-**

**1. Why are you not using toilet? /आप शौचालय का उपयोग क्यों नहीं कर रहे हैं?**

• Prefer open-defaecation due to fresh air & open fields /ताजी हवा और खुले खेतों के कारण खुले में शौच को प्राथमिकता

• Specified in religious scriptures /धार्मिक शास्त्रों में निर्दिष्ट है

• Financial constraints /वित्तीय बाधाएं

• Any other reason specify /कोई अन्य कारण निर्दिष्ट करें-

**2. Where do the male members go for defaecation? /पुरुष सदस्य शौच के लिए कहां जाते हैं?**

•open barren land /खुली और बंजर भूमि

•lush green lands /हरे भरे मैदान

•nearby nallah /पास का नाला

•Others /अन्य

**3. Where do the female members of the household go for defaecation? /घर की महिला सदस्य शौच के लिए कहां जाती हैं?**

•open barren land /खुली और बंजर भूमि

•lush green lands /हरे भरे मैदान

•nearby nallah /पास का नाला

•Others /अन्य

**4. Where do the kids of the household go for defaecation? /घर के बच्चे शौच के लिए कहाँ जाते हैं?**

•open barren land /खुली और बंजर भूमि

•lush green lands /हरे भरे मैदान

•nearby nallah /पास का नाला

•Others /अन्य

**5. Do you face any problems due to not having a toilet facility? What are they? /शौचालय की सुविधा नहीं होने के कारण क्या आपको कोई समस्या है? वे क्या हैं?**

• Any shameful incident with females of the house (harassment faced due to going in dark hours) /घर की महिलाओं के साथ कोई शर्मनाक घटना (अंधेरे घंटों में जाने के कारण उत्पीड़न)

• Time crunch & need to wake up too early /समय की कमी और बहुत जल्दी जागने की जरूरत है

• Illness related to bladder or stomach /मूत्राशय या पेट से संबंधित बीमारी

• Any accidents (snake, insects etc) /कोई भी दुर्घटनाएं (सांप, कीड़े आदि)

• Any other problem, specify /कोई अन्य समस्या, निर्दिष्ट करें-

**6. Do you think having a toilet can significantly improve above mentioned problems? If no, then how? /क्या आपको लगता है कि एक शौचालय होने से उपर्युक्त समस्याओं में काफी सुधार हो सकता है? यदि नहीं, तो कैसे?**

•Yes/ हाँ

•If No, why? /यदि नहीं, तो क्यों?

**7. Do you intend to pay more expenditure of money than the government's aid for the construction of toilets if it exceeds the subsidy? /क्या आप शौचालय के निर्माण के लिए सरकार की सहायता से अधिक धनराशि का भुगतान खर्च करने का इरादा रखते हैं यदि यह सब्सिडी से अधिक है?**

•Yes/ हाँ •No /नहीं

**8. In case there is no money from the government would you still construct a toilet on your own expense? /यदि सरकार के पास कोई पैसा नहीं है तो क्या आप अभी भी अपने खर्च पर शौचालय का निर्माण करेंगे?**

•Yes/ हाँ •No /नहीं

**9. According to you, how much money does it cost to construct a toilet? /आपके अनुसार, शौचालय बनाने में कितना पैसा लगता है?**

•0-10,000

•10,000- 20,000

• 20,000- 30,000

•30,000- 40,000

• 40,000- 50,000

• Don’t know /पता नहीं

**10.** **What problems do you incur with/while constructing a toilet? /शौचालय का निर्माण करते समय आप किन समस्याओं से जूझते हैं?**

• It takes a lot of time to supervise & construct /इसकी देखरेख और निर्माण में बहुत समय लगता है

• Quality of construction not as promised /निर्माण की गुणवत्ता के अनुसार वादा नहीं किया जाता है

• Needs regular maintenance & services /नियमित रखरखाव और सेवाओं की आवश्यकता है

• Contractor fills his own pocket /ठेकेदार अपनी जेब भरता है

• Lack of funds and resources /धन और संसाधनों की कमी

• No idea /पता नहीं

**11.** **How much money are you happily willing to pay for a good toilet facility in your house? /आप अपने घर में शौचालय की अच्छी सुविधा के लिए कितने पैसे देने को तैयार हैं?**

•0- 5,000

•5,000-10,000

•10,000-15,000

•15,000-20,000

•20,000-25,000

•nil/ शून्य

**12.** **What do you think of toilets which are low in cost and fast to construct? /क्या आप शौचालय के बारे में सोचते हैं जो लागत में कम होगा और जिसमें तेजी से निर्माण होगा?**

• It must be bad/faulty as such low cost is not possible /यह खराब / दोषपूर्ण होना चाहिए क्योंकि इतनी कम लागत संभव नहीं है

• Works best for us if someone can provide facility in less price/ यह हमारे लिए सबसे अच्छा होगा अगर कोई कम कीमत में सुविधा प्रदान कर सकता है

**13. Would you be interested in constructing a fast (in some hours) and easy to install toilet for your family? /क्या आप अपने परिवार के लिए एक तेज (कुछ घंटों में) और आसानी से स्थापित शौचालय का निर्माण करने में रुचि रखते हैं?**

• We do not want to get involved in construction process /हम निर्माण प्रक्रिया में शामिल नहीं होना चाहते हैं

• Sounds like fun and exciting /मजेदार और रोमांचक लगता है

**14. What material do you think toilets should be made of? /आपको क्या लगता है कि शौचालय किस सामग्री से बना होना चाहिए?**

• Local material used in construction like stone walls etc (suggest some) /इस क्षेत्र निर्माण में प्रयुक्त स्थानीय उपलब्ध सामग्री जैसे पत्थर की दीवारें आदि (कुछ सुझाव)

• Metal Sheets like steel and GI /स्टील और जीआई जैसी मेटल शीट्स

• Strong durable Plastic /मजबूत टिकाऊ प्लास्टिक

• Mud bricks / मिट्टी की ईंट

• Wood / लकड़ी

**15.**  **Where do you see advertisements for toilets? /आप शौचालय के विज्ञापन कहां देखते हैं?**

• Government Dispensaries/Hospitals /सरकारी औषधालय / अस्पताल

• Television/Mobile phones /टेलीविजन / मोबाइल फोन

• Village walls /गाँव की दीवारें

• Schools /विद्यालय

• Never heard of one / कभी किसी के बारे में नहीं सुना

**16.**  **What are your perceptions regarding using pit humus from the toilet? /शौचालय से प्राप्त खाद का उपयोग करने के बारे में आपकी क्या धारणाएं हैं?**

• I am willing to use / मैं उपयोग करने के लिए तैयार हूँ

• I am not willing to use /मैं उपयोग करने के लिए तैयार नहीं हूं

• I am willing to use but only for growing non- human consumption crops /मैं उपयोग करने के लिए तैयार हूं लेकिन केवल गैर-मानव उपभोग वाली फसलों को उगाने के लिए

**Appendix B: Figures used in Section 3**


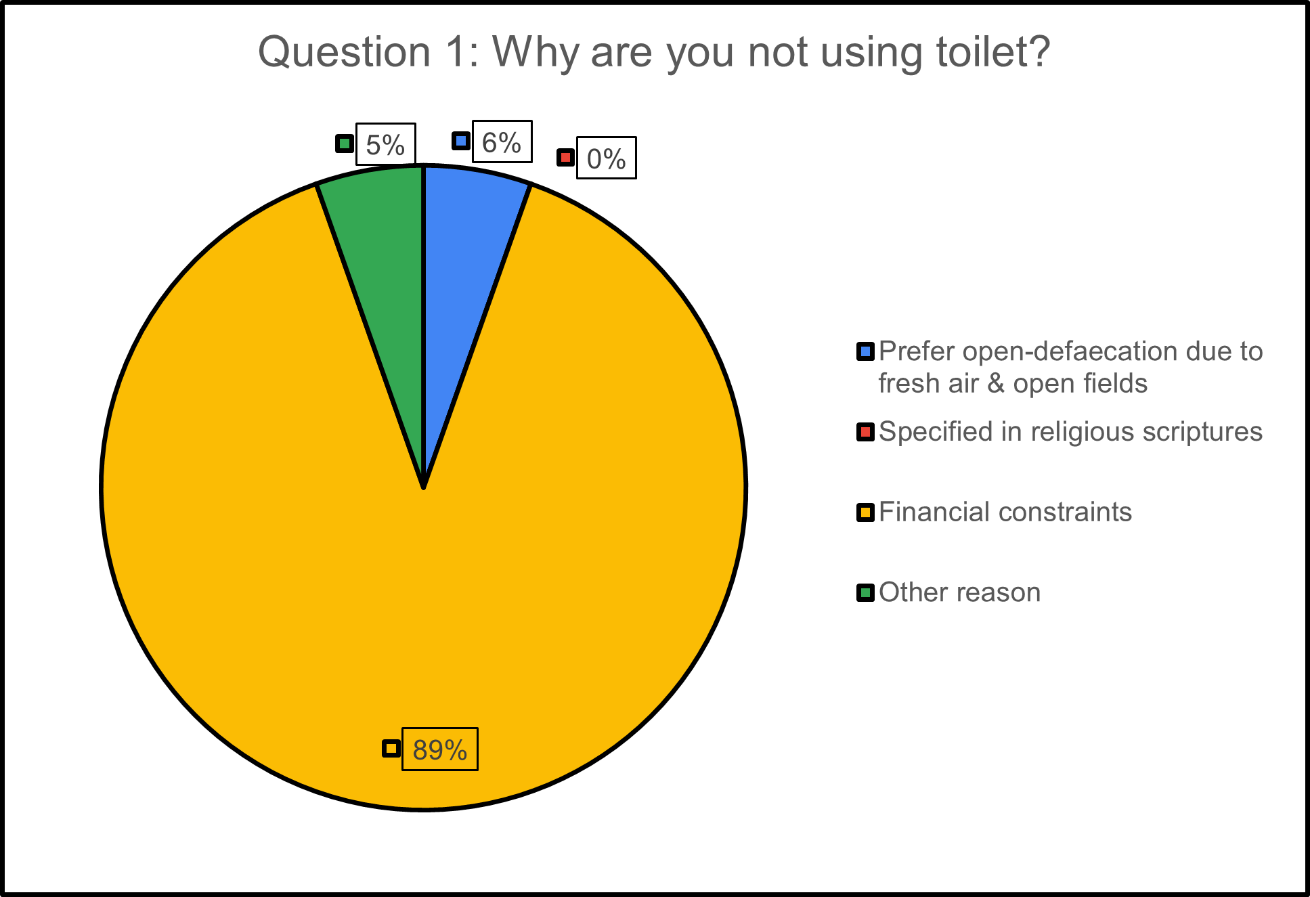


Figure B.1. Responses to Question 1 in Appendix A


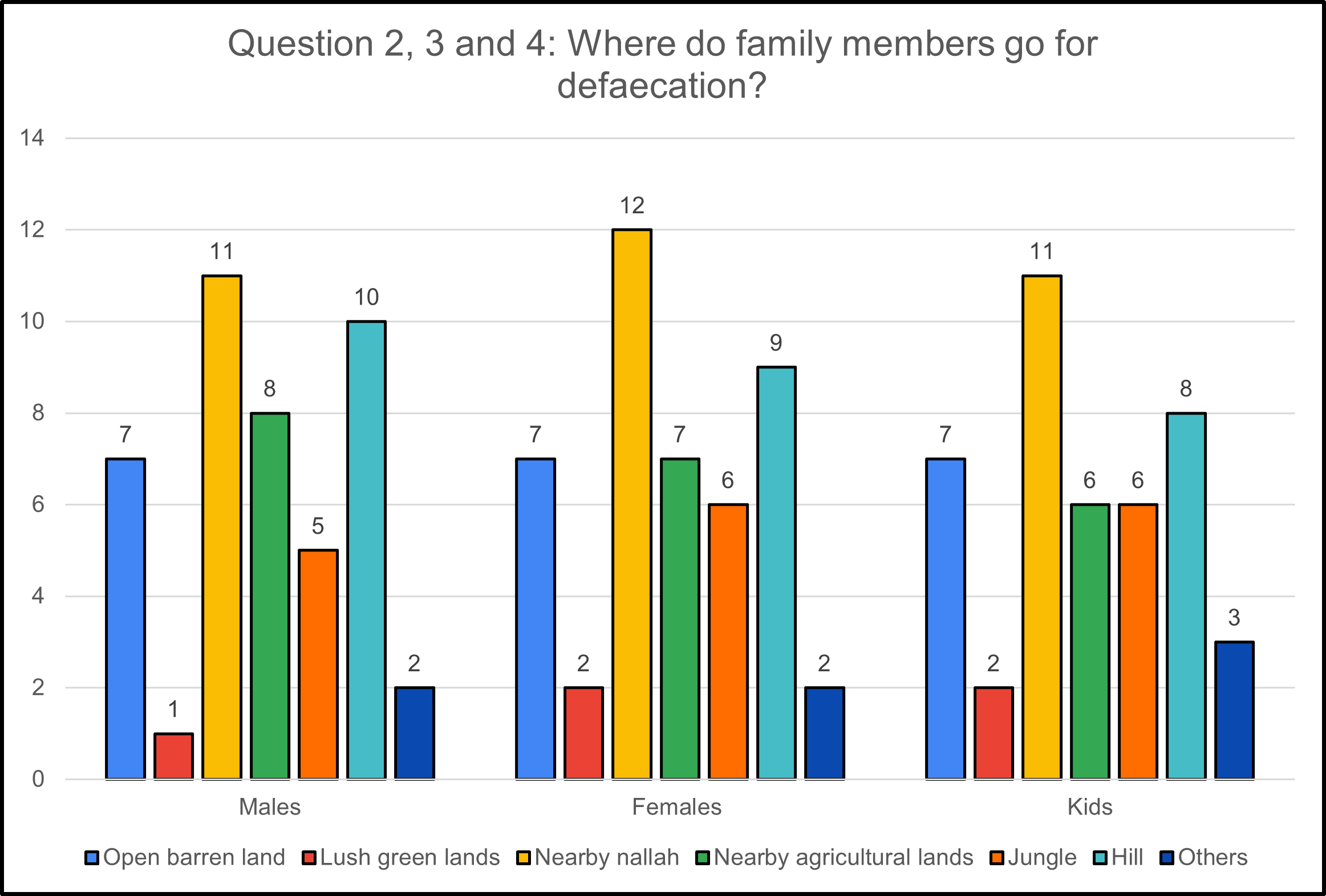


Figure B.2. Comparison of the responses to Questions 2, 3 and 4 in Appendix A


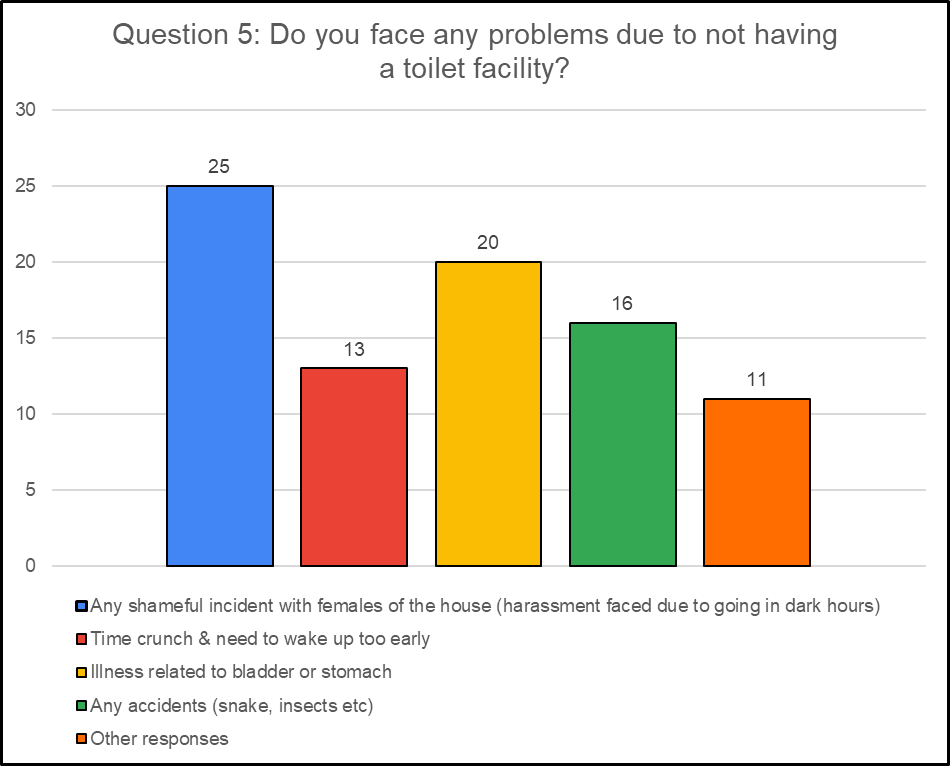


Figure B.3. Issues faced while opting for open defaecation as per Question 5 in Appendix A


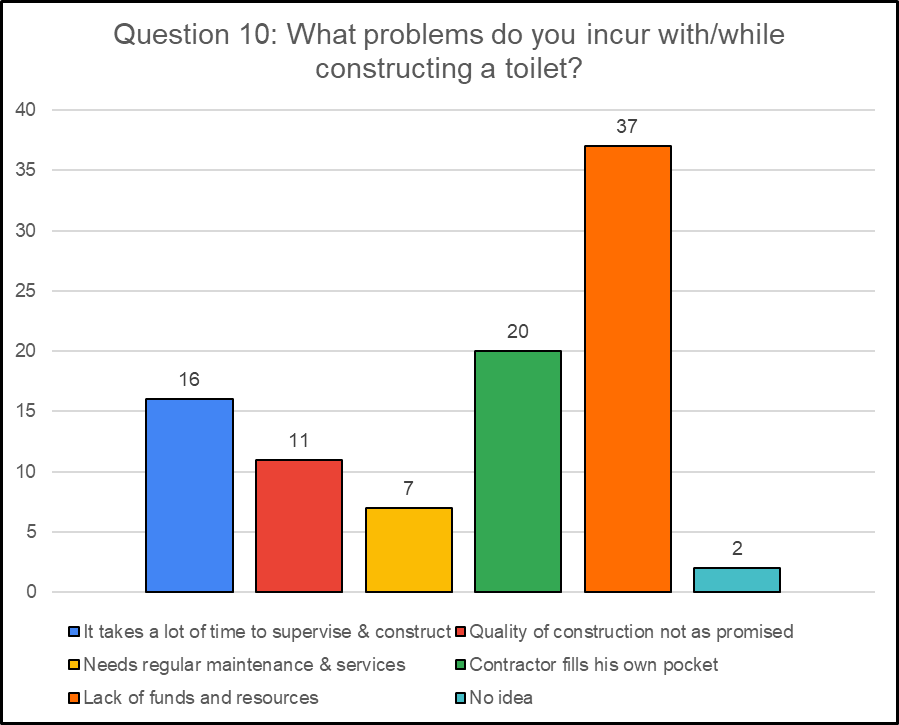


Figure B.4. Responses to Question 10 in Appendix 1


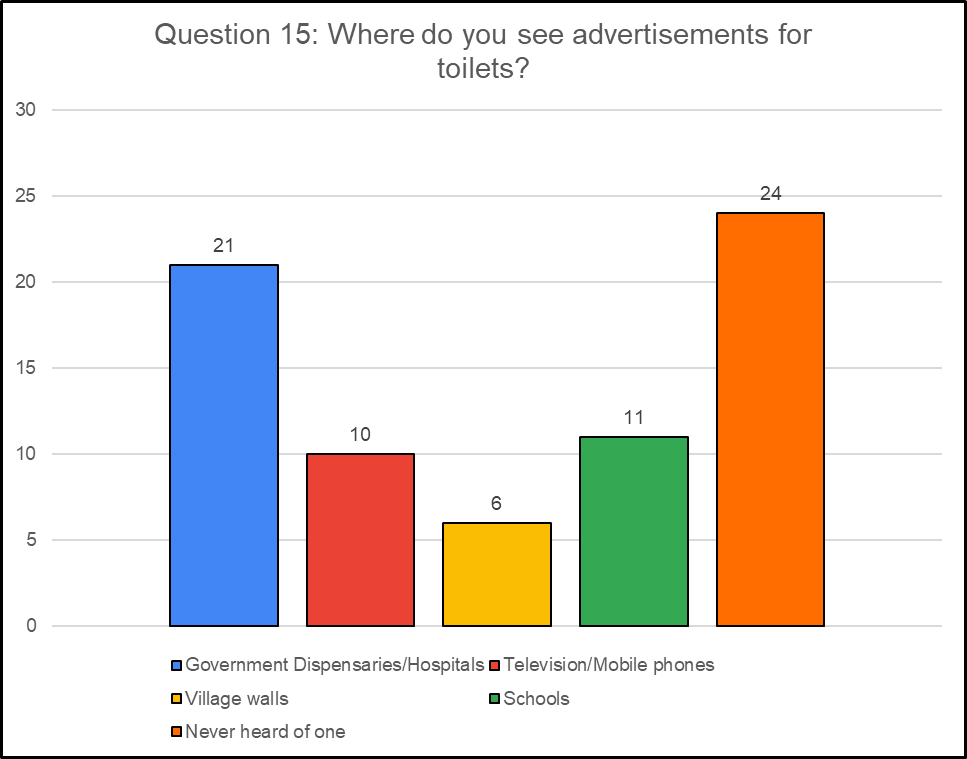


Figure B.5. Places where the toilet promotion advertisements are seen as a response to Question 15 in Appendix 1


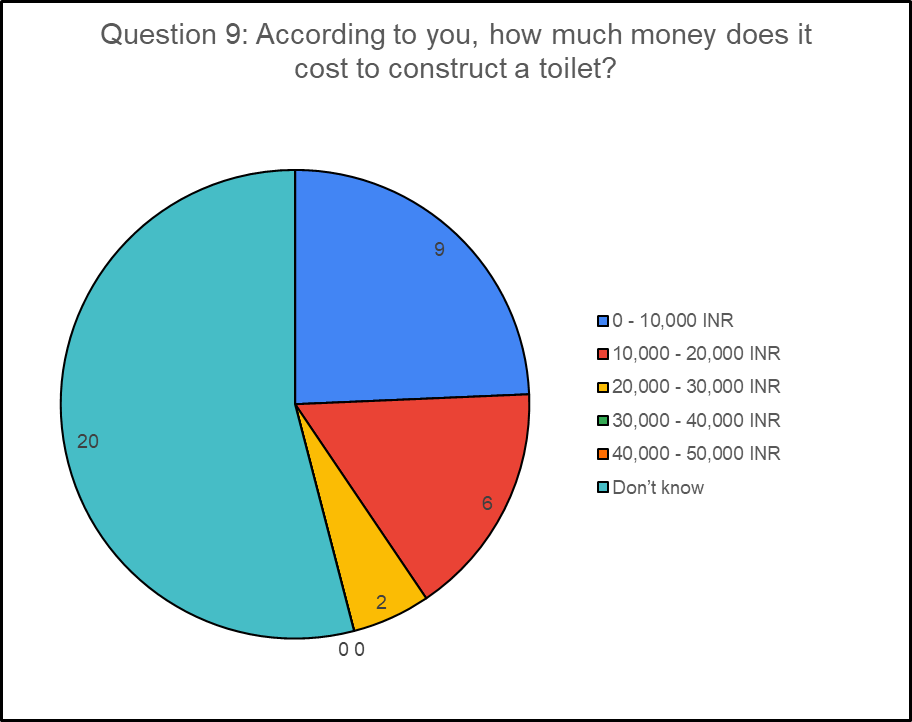


Figure B.6. Response to Question 9 in Appendix A, finding about the awareness of people regarding the cost of a toilet


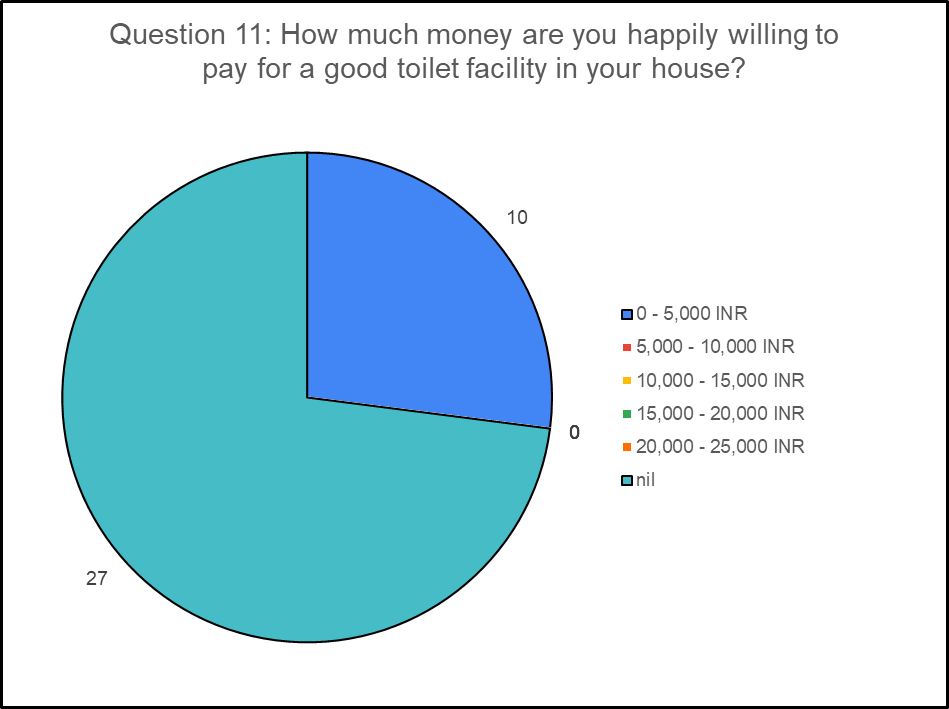


Figure B.7. Response to Question 11 in Appendix A to determine the state of mind of people to pay for the construction of a toilet at their own will


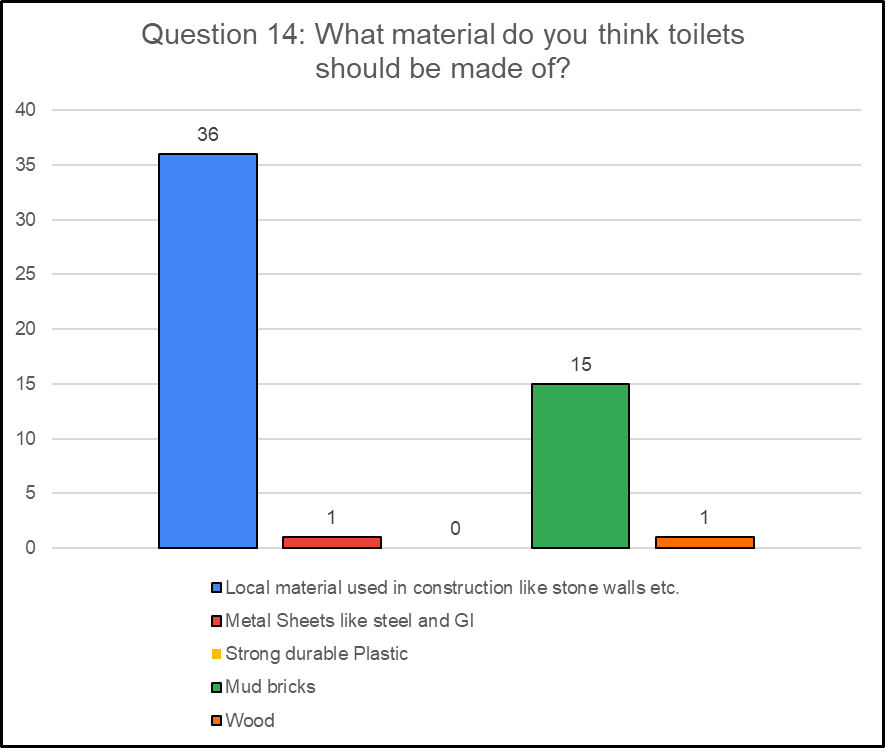


Figure B.8. Response to Question 14 in Appendix A to find out the preferred material for toilet construction


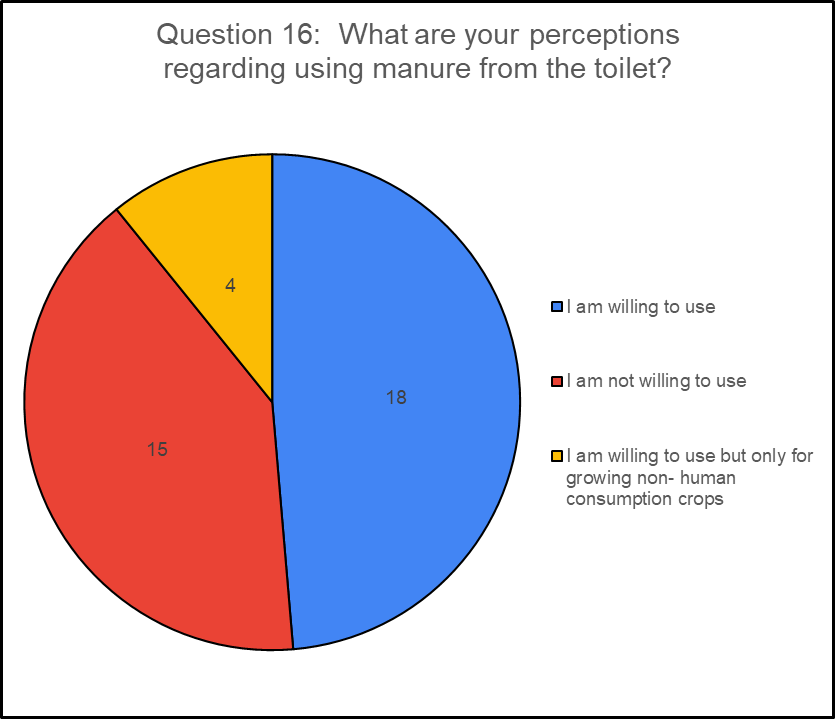


Figure B.9. Willingness to use the pit humus obtained from the toilet as a response to Question 16 in Appendix A **Appendix C: Results of the cross-table analysis as discussed in Section 3**

**Table C.1. Cross-table analysis between Question 9 and Question 12 (unit: households)**

|  | | **12. What do you think of toilets which are low in cost and fast to construct?** | |
| --- | --- | --- | --- |
|  |  | It must be bad/faulty as such a low cost is not possible | Works best for us if someone can provide a facility at less price |
| **9. According to you, how much money does it cost to construct a toilet?** | Don’t know | 4 | 16 |
|  | 0-10,000 INR | 1 | 8 |
|  | 10,000- 20,000 INR | 3 | 3 |
|  | 20,000- 30,000 INR | 0 | 2 |

**Table C.2. Cross-table analysis between Question 11 and Question 16 (unit: households)**

|  | | **16. What are your perceptions regarding using pit humus from the toilet?** | | |
| --- | --- | --- | --- | --- |
|  |  | I am not willing to use | I am willing to use but only for growing non-human consumption crops | I am willing to use |
| **11. How much money are you happily willing to pay for a good toilet facility in your house?** | Nil | 12 | 2 | 12 |
|  | 0- 5,000 INR | 3 | 1 | 6 |
|  | 5,000- 10,000 INR | 0 | 0 | 0 |
|  | 10,000- 15,000 INR | 0 | 1 | 0 |

**Table C.3. Cross-table analysis between Question 7 and Question 11 (unit: households)**

|  | | **11. How much money are you happily willing to pay for a good toilet facility in your house?** | |
| --- | --- | --- | --- |
|  |  | Yes (any value) | Nil |
| **7. Do you intend to pay more expenditure of money than the government's aid for the construction of toilets if it exceeds the subsidy?** | Yes | 7 | 3 |
|  | No | 4 | 23 |

**Table C.3. Cross-table analysis between Question 15 and Questions 11 and 12 (unit: households)**

|  | | **11. How much money are you happily willing to pay for a good toilet facility in your house?** | | **12. What do you think of toilets which are low in cost and fast to construct?** | |
| --- | --- | --- | --- | --- | --- |
|  |  | paying something | paying nothing | Works best for us if someone can provide a facility at less price | It must be bad/faulty as such a low cost is not possible |
| **15. Where do you see advertisements for toilets?** | Yes, seen an advertisement | 2 | 14 | 13 | 3 |
|  | Nope, never saw an advertisement | 9 | 12 | 16 | 5 |
